# Pediatric pemphigus herpetiformis treated with rituximab

**DOI:** 10.1016/j.jdcr.2024.09.009

**Published:** 2024-10-28

**Authors:** Mira Hamed, Judith Krausz, Michael Ziv, Eran Cohen Barak

**Affiliations:** a Dermatology Department, Emek Medical Center, Afula, Israel; b Department of Pathology, Emek Medical Center, Afula, Israel; c Bruce and Ruth Rappaport Faculty of Medicine, Technion, Haifa, Israel

**Keywords:** autoimmune bullous skin diseases (AIBDs), pediatric pemphigus, pemphigus herpetiformis (PH), rituximab

## Introduction

Pediatric pemphigus is a group of autoimmune bullous diseases in the pediatric population, representing diagnostic and therapeutic challenge. Pemphigus herpetiformis (PH) is an exceptionally rare form of pemphigus, with limited data regarding its course. This report highlights a unique case of a 5-year-old girl who initially presented with blistering and itching rash. A diagnosis of PH was established based on clinical, histologic, and immunohistochemical findings. Despite resistance to first-line therapy, successful treatment was achieved using rituximab, marking it as the first documented case of its kind in the pediatric population within the literature.

## Case report

**Fig 1:**
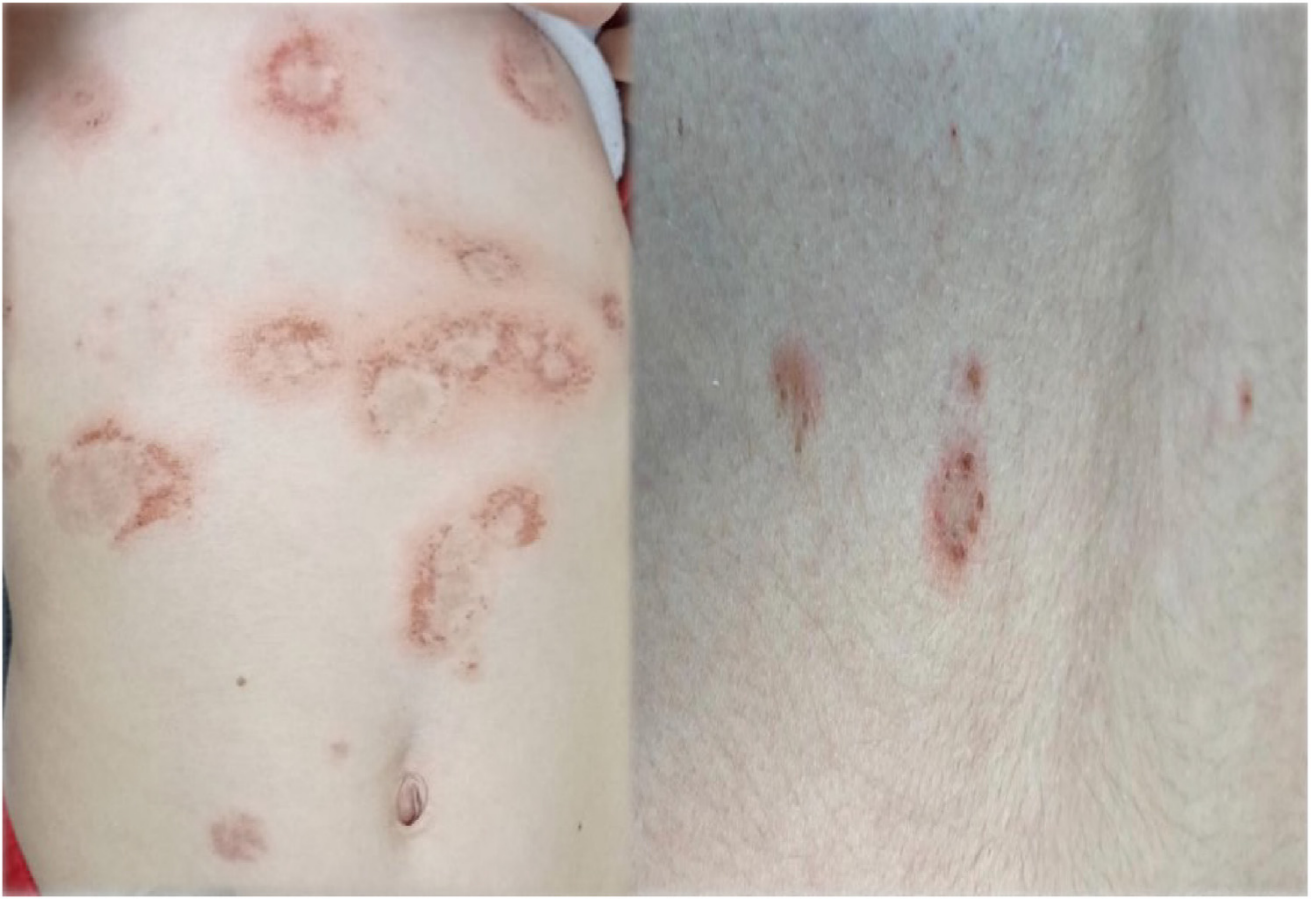
Annular erythematous plaques, along with multiple erosions over the torso

A 5-year-old girl of Moroccan Jewish descent was referred due to 2-week history of a pruritic rash over her head, trunk, and lower limbs. Physical examination revealed annular erythematous plaques, lined by vesicles and erosions over her scalp, trunk, and limbs. Nikolsky's sign was negative. Notably, there was no mucosal or genital involvement (Fig 1).

**Fig 2:**
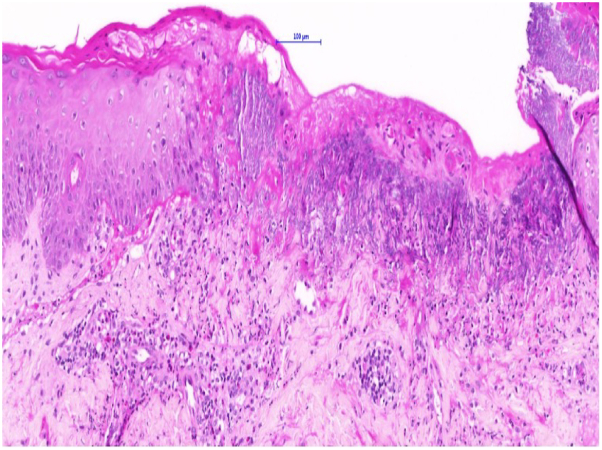
Biopsy (H&E stain) from the left buttock showing eosinophilic spongiosis. *H&E*, Hematoxylin and eosin.

**Fig 3:**
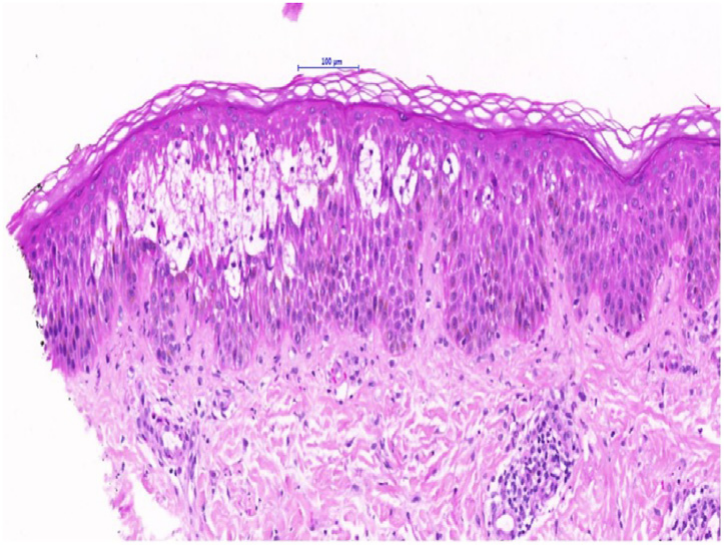
Biopsy (H&E stain) from the right calf showing focal intraepidermal blistering with acantholysis.

The differential diagnosis of autoimmune blistering diseases was considered. Skin biopsies were obtained, revealing eosinophilic spongiosis and focal intraepidermal blistering with acantholysis and perivascular lymphocytic infiltration (Figs 2 and 3).

Direct immunofluorescence testing revealed a net-like linear deposition of IgG between keratinocytes, with C3 being negative. Indirect immunofluorescence testing was positive on monkey esophagus at a titer of 1:10. Clinical measurement of Dsg 1 levels was not performed.

Based on the clinical presentation, histopathology, and immunofluorescence findings, a diagnosis of pediatric pemphigus herpetiformis was made.

Prednisolone at a dosage of 1 mg/kg was initiated along with the dapsone at a dosage of 1.5 mg/kg/day, which resulted in complete remission. However, attempts to taper corticosteroid dosage down below 0.5 mg/kg/day resulted in disease relapse. As a result, the patient was treated with 2 sequential doses of rituximab 375 mg/m^2^, administered 2 weeks apart, which led to complete response, under minimal dose of glucocorticoids (prednisolone 3 mg/day).

To the best of our knowledge, this is the first reported case of pediatric pemphigus herpetiformis treated successfully with rituximab.

## Discussion

Autoimmune bullous skin diseases are exceptionally rare in children, which poses challenges in diagnosis and treatment. Practitioners must consider autoimmune bullous skin diseases in the differential diagnosis for pediatric patients with persistent erosions on the skin and/or mucosa. The spectrum of potential differential diagnoses is broad, including conditions like bacterial infections (eg, bullous impetigo), viral or fungal infections, drug eruptions, or staphylococcal scalded skin syndrome. The exact incidence of autoimmune bullous skin diseases in the pediatric population remains uncertain. In the United States, dermatitis herpetiformis is the most common while linear IgA bullous dermatosis is prevalent in Singapore.^1^^,^^2^

PH, a term coined in 1975, is estimated to account for approximately 6% of pemphigus cases in adult populations.^3^^,^^4^ The condition is known to manifest across a wide age range, from infancy to 92 years, with the typical onset occurring during the fifth or sixth decade of life.^5^^,^^6^ To date, only 11 pediatric PH cases have been reported (as shown in Table I), with a mean age of 7 years.

**Table I:** Reported cases of pediatric pemphigus herpetiformis

Reference	Age/gender	Skin	Mucosal involvement	Pruritus	Histopathology	Therapy	Response (CD-controlled disease. CR- complete remission)
Our case/Israel	5/F	Annular erythematous plaques with vesicles and erosions on scalp, trunk, and limbs.	No	Yes	Eosinophilic spongiosis and focal intraepidermal blistering with acantholysis and perivascular lymphocytic infiltration DIF-IgG (intercellular) IIF-1:10	Prednisolone (1 mg/kg) and dapsone (1.5 mg/kg/d) induced remission. Corticosteroid taper below 0.5 mg/kg led to relapse. Sequential rituximab doses achieved complete response with minimal dose of corticosteroid.	CD
Hayder et al^7^ 2022/Tunisia	4/M	Annular erythematous plaques on chest and thighs, hyperpigmented patches, crusted erosions on scapular area, multiple blisters on left leg with a herpetiform pattern, and tense vesicles on both soles Nikolsky’s (−)	No	Yes	Intact bulla: "tomb-stone" cleft in basal and suprabasal layers. Mixed inflammatory infiltrate of neutrophils and eosinophils in epidermis. Edema of dermis with mixed spongiosis and perivascular deposition of lymphocytes DIF-IgG and C3 (intercellular) IIF-NA ELISA-Dsg1 (135UI/ml) Dsg3-negative	Topical betamethasone dipropionate 0.05% improved. Relapse after 3 mo, started dapsone (1 mg/kg/daily, then increased to 2 mg/kg/daily). Remarkable improvement.	CD
Peterman et al^8^ 2017/United states	2/F	Eczematous plaques and blisters erupted on her face, hands, feet, and trunk. Erosion of the labia minora.	No	Yes	Intraepidermal vesicle with neutrophils and acantholytic cells. Eosinophilic infiltrate in epidermis and dermis DIF-IgG and C3 (intercellular) IIF-Intermediate, predominantly negative ELISA-Dsg1	Topical clobetasol ointment, cephalexin brought partial relief. Rash flared postantibiotic taper. Switched to dapsone (1.5 mg/kg/d), achieved complete remission	CD
Akoglu et al^9^ 2017/Turkey	9/M	Annular and herpetiform vesicles and bullae (0.5-2 cm) over faint erythematous background on trunk and extremities Nikolsky’s (−)	No	Yes	Epidermal spongiosis, intraepidermal cleft, acantholytic cells in bulla, papillary dermis inflammation with eosinophils. DIF-IgG and C3 (intercellular) ELISA-Dsg1 + (1:100)	Initiated systemic therapy: methylprednisolone 1 mg/kg, cetirizine 5 mg/d, and betamethasone cream twice daily. Lesions improved, lowered steroid to 0.5 mg/kg. New widespread lesions appeared, raised steroid to 1 mg/kg. Added methotrexate (10 mg/wk) and folic acid (5 mg), achieved regression in 3 mo. Gradually reduced methylprednisolone to 0.25 mg/kg. After lost follow-up, resumed steroids, then tapered. No relapses observed for 8 mo without treatment.	CR
Schoch et al^5^ 2015/United states	Neonate/M	Crateriform erosions on both hands and feet	No	-	Focal intraepidermal acantholysis, prominent eosinophilic and neutrophilic exocytosis DIF-C3 (intercellular)	None	CR at 3 wk of age with milia
Leithauser et al^10^ 2013/United states	9/M	Round and annular, eroded, crusted, erythematous plaques on legs, arms, back, chest, and abdomen with closely set 2- to 5-mm round vesicles	Not available	Yes	Neutrophilic and eosinophilic spongiosis with intraepidermal vesicles, and many neutrophils, eosinophils, and lymphocytes in the superficial dermis DIF-IgG and C3 (epidermal cells surface) IIF (monkey esophagus)-negative	Various treatments such as dapsone, mycophenolate mofetil, azathioprine, rituximab, doxycycline, nicotinamide, and erythromycin, with no significant improvement, prednisone and oral methotrexate (15 mg/wk) were initiated, resulting in gradual improvement and complete remission	CR
Moutran et al^11^ 2011/Lebanon	6/F	Vesicles and bullae on annular, polycyclic, erythematous plaques, with residual hyperpigmentation on the trunk, face, and extremities.	No	Yes	Acantholysis affecting the middle and superficial layers of the epidermis with neutophilic infiltration DIF-IgG and C3 (intercellular)	Treated with prednisone (10 mg/d, 0.3 mg/kg/d), partial regression, relapses with dose reduction. Dapsone initiated (2 mg/kg/d), prednisone tapered, stopped after 3 mo. Maintained dapsone (2 mg/kg/d) for 24 mo, no relapses.	CD
Hocar et al^12^ 2011/Morocco	12/M	Vesicular, bullous lesions with clear or purulent contents, erosive arciform plaques, and crusted lesions. Predominant on back, buttocks, chest, abdomen, legs, and arms Nikolsky’s (−)	No	Yes	Intraepidermal bulla with rare acantholytic cells, eosinophils, and neutrophils. Lower epidermis showed eosinophilic spongiosis and focal acanthosis. Inflammatory infiltrate of eosinophils and neutrophils around some blood vessels in superficial and reticular dermis. DIF-IgG and C3 (intercellular) IIF-1:200	Dapsone 2 mg/kg daily induced remission. After 2 mo, prednisone (2 mg/kg) treated new vesicles effectively. Lesions regressed within 4 wk. Prednisone dose gradually lowered based on improvement. After a year, skin involvement controlled with 10 mg daily prednisone.	CD
Duarte et al^13^ 2010/Brazil	5/F	Annular plaques grouped vesicles in the periphery, central ulceration, and crusts on trunk, lower limbs, face, external genitalia, and buttocks. Erythematous lesions and tense grouped blisters on upper limbs.	No	Yes	Subcorneous blisters with rare acantholytic cells and numerous polymorphonuclear cells.The adjacent epidermis displayed multiple foci of spongiosis and eosinophilic exocytosis DIF-IgG and C3 (intercellular)	Dapsone (50 mg/d) ineffective for 10 d. Exfoliative dermatitis developed. Prednisone (20 mg/d) + dapsone initiated, remission achieved. Corticosteroids tapered, vesicles recurred. Azathioprine (50 mg/d) + increased prednisone, then withdrawn after remission.	CR
Huhn et al^14^ 1996/Canada	14/F	Symmetrical polymorphous rash consist of erythematous macules, pink papules mixed with clear and cloudy vesicles, and irregular ulcerated and crusted lesions arranged in a herpetiform group	No	Yes	1st biopsy: Mild epidermal hyperplasia with focal spongiotic areas containing scattered neutrophils, lymphocytes, and eosinophils. Superficial perivascular infiltrate in papillary dermis. 4th biopsy: Acantholytic cells with neutrophils in mid-epidermal cavities. No papillary dermal microabscesses. DIF: IgG and C3 (intercellular) IIF-negative	Initiated prednisone 40 mg/d, tapering by 10 mg every 5 d. 95% clearance in 10 d. Relapsed on cessation, responded to resumed and tapered prednisone. Stable on low dose.	CD
Adams et al^15^ 2009/United states	10/F	Hypopigmented macules, crusted papules, annular plaque, and tense fluid blisters on lower limb	Not available	Yes	Initial biopsy: Eosinophilic spongiosis, necrotic keratinocytes, no acantholysis. Subsequent biopsy: Intraepidermal acantholytic vesicles with eosinophilic and neutrophilic spongiosis. DIF-Positive IgG (epidermal cell surface) IIF-1:160. ELISA-Dsg 1: Slightly elevated.	Patient decline treatment	-
Maggiore et al^16^ 2019/Italy	8/F	Erythematous-edematous plaques, some arciform, with excoriated and hemorrhagic crusts on limbs and back	No	Yes	Intraepidermal vesicular dermatitis with mainly neutrophils and rare eosinophils, and sporadic acantholytic cells. Surrounding epidermis: neutrophilic and focally eosinophilic spongiosis DIF: IgG intercellular, C3 IFF (monkey esophagus): 1:40 ELISA-Dsg 1	Prednisone (1 mg/kg/d) initiated, tapered, with dermatitis recurrence. Added dapsone (2 mg/kg/d), ineffective during prednisone taper. Azathioprine (2 mg/kg/d) started, aiming to reduce ongoing steroid dose for lesion remission and disease control.	CD

*Dsg*, Desmoglein.

Clinically, PH shares similarities with dermatitis herpetiformis, yet it displays distinctive pathologic and immunohistochemical features characteristic of pemphigus. Almost all cases presented with annular erythematous plaques, occasionally accompanied by few blisters or erosions, with no predilection site. Mucosal involvement was absent, and only 1 case showed genital involvement. Histopathologic findings revealed spongiosis with either eosinophilic, neutrophilic, or both types of infiltration in all cases, with a slightly more predominant eosinophilic pattern compared to the neutrophilic pattern. Immunologically, direct immunofluorescence was positive in all cases, and indirect immunofluorescence disclosed.

Pruritus emerges as a prominent clinical feature in PH. Among the cases reviewed, 11 out of 12 reported experiencing an intense pruritic rash, which could be explained by the increased infiltration of dermal IL31^+^ cells and IL-31RA^+^ cells, accompanied by an increased number of dermal eosinophils and basophils. This also implies for potential pathways implicated in the manifestation of pruritus in PH.^17^

The underlying pathomechanism remains elusive, possibly involving a combination of severe inflammation and autoimmune processes targeting Dsg 1. However, reports indicate involvement of Dsg 3 and desmocollin 1, 2, and 3.^18^ Additionally, drug-induced PH was also reported.^9^

PH is known for its indolent clinical course and favorable response to dapsone, either as monotherapy or in combination with corticosteroids.^6^ Additionally, it is characterized by the potential to transition into pemphigus foliaceus or pemphigus vulgaris. In our case, the patient showed resistance to first-line therapy, prompting the initiation of rituximab treatment, which resulted in successful disease control. This outcome is consistent with a systematic review conducted by Patel et al,^19^ which demonstrated predominantly positive responses to rituximab treatment in other pediatric pemphigus cases, mainly pemphigus vulgaris or foliaceous. The optimal regimen is currently controversial. For adolescents, 2 commonly regimens are employed: the rheumatoid arthritis protocol, comprising of 2 1000 mg infusions with a 2-week interval, and the lymphoma protocol, involving 4 weekly infusions of 375 mg/m^2^. For children, potential regimens include 700/500/375 mg/m^2^ with a 2-week interval.

In conclusion, our report has focused on pediatric pemphigus herpetiformis and presented the first case of successful rituximab treatment. Nonetheless, further investigation into pemphigus cases among children is warranted to gain a deeper understanding of the underlying (Supplementary Table I, available via Mendeley at https://doi.org/10.17632/wr9fvf7fkw.1).

## Conflicts of interest

None disclosed.
